# Working under the 2021 Heat Dome: A Content Analysis of Occupational Impacts Mentioned in the Canadian Media

**DOI:** 10.3390/healthcare11172423

**Published:** 2023-08-30

**Authors:** Emily J. Tetzlaff, Nicholas Goulet, Melissa Gorman, Leonidas G. Ioannou, Glen P. Kenny

**Affiliations:** 1 Human and Environmental Physiology Research Unit, School of Human Kinetics, Faculty of Health Sciences, University of Ottawa, 125 University Private, Ottawa, ON K1N 6N5, Canada; etetz085@uottawa.ca (E.J.T.);; 2 Climate Change and Innovation Bureau, Healthy Environments and Consumer Safety Branch, Safe Environments Directorate, Health Canada, 269 Laurier Avenue West, Ottawa, ON K1A 0K9, Canada; melissa.gorman@hc-sc.gc.ca; 3 Behavioural and Metabolic Research Unit, School of Human Kinetics, Faculty of Health Sciences, University of Ottawa, 200 Lees Avenue, Ottawa, ON K1N 6N5, Canada; 4 Department of Automatics, Biocybernetics and Robotics, Jožef Stefan Institute, 1000 Ljubljana, Slovenia; ioannoulg@gmail.com; 5 Clinical Epidemiology Program, Ottawa Hospital Research Institute, Ottawa, ON K1Y 4E9, Canada

**Keywords:** heat stress, heat strain, occupational health and safety, workplace exposure, climate change, worker protection

## Abstract

Extreme heat events directly impact worker health and cause additional cascading and transitional workplace impacts. However, current investigations on these impacts often rely on specific datasets (e.g., compensation claims, hospitalizations). Thus, to continue to work towards preventing and mitigating the occupational risks posed by extreme heat events, this study aimed to explore the occupational impacts of the 2021 Heat Dome in Canada using a qualitative content analysis method on a news-based dataset. A systematized review of news articles published before, during, and after the 2021 Heat Dome was conducted on academic (*n* = 8) and news (*n* = 5) databases, along with targeted grey literature. Two researchers qualitatively coded the articles in NVivo for occupational impacts or references mentioned within the articles. Overall, 52 different occupations were identified as being impacted by the 2021 Heat Dome. Impacts were diverse and ranged from work cancellations or delays to work modifications and reports of heat-related illnesses. The 2021 Heat Dome impacted the health and safety of many occupational groups and provided new insights into the expanding impacts that extreme heat events can have on the Canadian workforce. With climate projections showing a growing trend of more hot days and intense heat waves in Canada, addressing these concerns should be a critical priority.

## 1. Introduction

Extreme heat poses a significant occupational health and safety (OHS) challenge for workers across multiple sectors, including the military, construction, mining, agricultural, manufacturing, and metal industries [1,2]. Workers in these sectors are often required to perform labour-intensive tasks while donning personal protective equipment in extreme ambient conditions [3,4,5], which can result in heat-related illnesses and injuries (e.g., heat stroke, acute kidney injury, adverse cardiovascular events) [1,6,7] and productivity decrements [1], especially when prolonged work in the heat is performed over consecutive days [8]. Studies have demonstrated a cumulative effect of successive workdays in the heat on thermoregulatory function [8]. These next-day effects not only result in lower heat tolerance and higher risk for heat-related illnesses but may result in worker fatigue and diminished work performance on subsequent days, leading to a rise in occupational accidents [1,9]. Due to the increase in the frequency, duration, and intensity of climate change-induced extreme heat events (or heat waves) [10,11], the challenges posed by occupational heat are becoming increasingly complex and are further exacerbating the threat posed by heat to these workers [2,12].

In addition to this aggravated threat to already traditionally heat-exposed labour groups, the risk of occupational heat stress is now extending to other sectors that may be less prepared to manage and mitigate the heat (e.g., workers in unairconditioned indoor environments, less physically demanding occupations such as office staff, retail workers) [13,14]. However, the existing literature primarily focuses on outdoor workforces (e.g., agriculture, construction, electric utilities) or those working around process-generated heat (e.g., underground mining, foundry workers) [12]. Our understanding of how prolonged periods of elevated ambient temperature may impact a broader array of occupations is therefore limited. Traditionally, such investigations have been conducted using sources like workers’ compensation claims data or hospitalizations to identify workers at higher risk of heat-related impacts (e.g., heat-related illness) [12,15,16,17,18,19,20,21]. However, it is well-established that heat-related illnesses and injuries, as well as indirect consequences of heat (e.g., heat-related accidents including traumatic injuries (i.e., fractures [7])), are consistently underreported across all sectors [12]. Therefore, it is speculated that current projections significantly underestimate workers’ risk when performing physically demanding work in hot environments or during extreme heat events. Thus, exploring other data sources and approaches to provide a more holistic understanding of the workforces impacted by high-temperature extremes is warranted.

One strategy employed in other disciplines has been harnessing news media as a source of information because it is a timely resource of thematic content [22,23,24,25]. In the context of extreme heat, the media serves as a medium for communicating heat warnings and public health messaging [25] and a means for sharing stories of impacts on services, systems, and people [26]. In Canada, Environment and Climate Change Canada (a federal government agency) issues extreme heat warnings which are accompanied by heat-health messages developed jointly with Health Canada (another federal agency) [27]. Once issued, these warnings and associated heat-health messages are often disseminated for widespread public awareness via media coverage [27]. Although OHS is primarily provincially and territorially regulated in Canada, a portion of the Canadian workforce falls under the OHS jurisdiction of the federal government [28], and thus the federal heat-health messages include the following two work-related statements: “*Extreme heat affects everyone. The risks are greater for young children, pregnant women, older adults, people with chronic illnesses and people working or exercising outdoors*”, and “*Outdoor workers should take regularly scheduled breaks in a cool place*” [27]. Despite the inclusion of these occupational heat-health statements, no study to date has specifically explored media coverage as a medium for understanding how the occupational impacts of an extreme heat event are communicated.

Therefore, we conducted a systematized review and content analysis of media articles published on one of the most extreme heat events in Canadian history—the 2021 Heat Dome (see context summary below)—to identify the occupations, including emerging at-risk sectors, reported in the media to have been impacted by the 2021 Heat Dome in Canada. Furthermore, we sought to explore the nature of the impacts faced by workers and workplaces during the extreme heat event, with the aim of employing this information to help identify strategies for managing occupational heat stress and to enhance the health and safety of those working in hot environments.

## 2. Materials and Methods

### 2.1. Context: The 2021 Heat Dome

Between 24 June and 4 July 2021, the ‘2021 Heat Dome’ (an extreme heat event) engulfed western Canada, primarily affecting British Columbia, Alberta, and regions of the Northwest Territories, Yukon, Saskatchewan, Manitoba, and northwest Ontario [29]. During this period, the western region achieved over 100 all-time heat records, including Canada’s highest temperature in Lytton, British Columbia (49.6 °C (121.3 °F)) [30]. Overall, the region ranged between 11 and 19 °C (51.8–66.2 °F) above average during the ten-day event, along with higher-than-average nighttime temperatures. The unprecedented intensity of the extreme heat event was attributed to the impact of climate change [31]. This extreme heat event claimed the lives of 619 individuals in British Columbia [32] and 66 more in Alberta [33] due to the impacts of heat (e.g., hyperthermia) [32]. Nearly all deaths in British Columbia were reported to have occurred indoors in personal residences; none of the deaths reported were related to occupational work [32].

### 2.2. Selection Criteria and Dataset

This project draws on a subset of data collected for a broader analysis of the 2021 Heat Dome in Canada [26]. A systematized review of digitized media content (e.g., newspaper articles, blogs, newsletters, community bulletins, municipal meeting minutes, public health unit posts, radio broadcasts, and TV transcripts) was conducted using eight academic databases (Medline, Embase, CAB Abstracts/Global Health, Agricola, FSTA, EconLit, PsycINFO, and Scopus) and five subscription news databases (ProQuest Canadian Major Dailies, Business Source Elite, NewsDesk, Factiva, and Eureka). In addition to the database searches, a list of targeted public and non-profit organization websites (see specifics below) was created for each of Canada’s thirteen provinces and territories, along with a list of national sites, to ensure a comprehensive grey-literature search of traditional (e.g., electronic newspapers) and non-traditional media sources (e.g., health agency authored news articles). The search strategy was developed in consultation with a Research Librarian, and the final search underwent review by a secondary Librarian before database translation (see supplemental file in [26] for further details). Articles published between 1 June 2021 and 26 February 2022 (date of search) were eligible for inclusion. The search strategy therefore aimed to capture all press published after the forecasted extreme weather alert, during the heat event, and post-event. Articles from the broader study dataset that included content related to workers/workplace settings were identified as information-rich cases for this secondary analysis. The resulting dataset included 520 articles published in Canada, representing 18% of the larger dataset.

### 2.3. Coding the Data

During full-text screening for the broader impact analysis, the authors created a working codebook of concepts, positive indicators, and textual examples inductively related to workplace heat impacts. The codebook included two layers of codes, including inductive (data-driven) and deductive (concept-driven) codes. The primary coding focused on inductively categorizing the media article content by each occupation identified within the coverage. The secondary coding then deductively characterized the type of occupational impact referenced in the media as direct, cascading, or translational. For this study, direct impacts were coded as effects reported in the press resulting from the heat, such as workers suffering heat-related illnesses. Cascading (or indirect) impacts were coded as knock-on effects from the heat described in the media, such as increased stress from an upsurge in work demand responding to the direct impact (e.g., paramedics responding to heat-related illness calls) [1,9]. Lastly, translational impacts were coded as the consequences of heat mitigation and adaptation efforts [9]. For example, media reports of workplaces experiencing increased costs associated with extra staffing to facilitate shorter shift rotations during extreme heat or work cancellation due to the heat causing a loss of income and potentially job security (e.g., seasonal pickers). It is important to note that an article could be assigned to multiple codes, as the article content could have discussed an array of impacts from the 2021 Heat Dome. This codebook was circulated to all authors for review and agreement. Modifications were made to collapse and expand codes as needed. The codebook and articles were uploaded to NVivo (Release 1.6.2, QSR International, Burlington, NJ, USA). A trial coding of 500 randomly selected articles (10% of identified articles) was then completed independently by E.J.T. and N.G. to ensure consistency (reliability) and validity of the category definitions. The authors achieved a kappa coefficient of 0.80 for this sub-analysis, indicating a ‘very good/excellent’ agreement [34]. All articles were then fully coded. 

### 2.4. Content Analysis

After coding was complete, the characteristics of the included documents and extracted data (coded findings) were analyzed using a series of NVivo query functions (e.g., dates of publication, publication type, word frequency). A qualitative content analysis method for systematically describing the meaning of the data was then used [35,36]. The authors then met to discuss the data to agree on the broader themes and concepts. 

## 3. Results

### 3.1. Occupational Groups Impacted by the Heat Dome

Among the articles that mentioned occupations impacted by the 2021 Heat Dome (*n* = 705), 52 occupational groups were identified (Table 1).

### 3.2. Workplace Impacts of the 2021 Heat Dome

**Figure 1:**
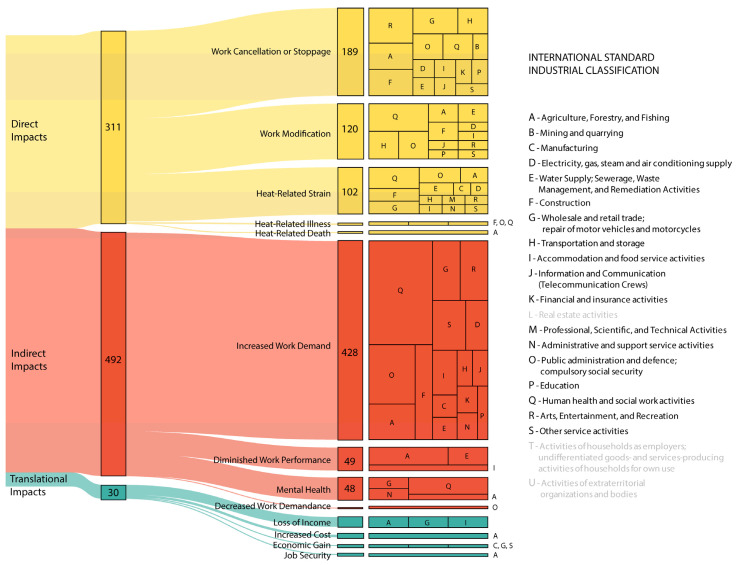
Direct, cascading, and translational workplace impacts of the 2021 Heat Dome as portrayed in the Canadian media. The values presented with each impact category and sub-group indicate the number of articles that included content related to that impact. Note: there were no reported impacts for the occupations associated with the International Standard Industrial Classifications *L*, *T*, *U*; these classifications are represented with light grey font. As the articles were coded to multiple occupational impact categories where applicable, the indicated count values are greater than the total number of articles (*n* = 705). Detailed information can be found in Table S1.

Within the media coverage of the 2021 Heat Dome, the occupational impacts mentioned were categorized as direct (*n* = 311), cascading (indirect) (*n* = 492), and translational (*n* = 30) (Figure 1, Table S1). Note: As the articles were coded to multiple occupational impact categories where applicable, the indicated count values are greater than the total number of articles, *n* = 705. Quotations from the news articles analyzed are used throughout the following sections (as indicated using numeric formatting in square brackets) to provide evidence and supplement the impact classifications and impact types identified

#### 3.2.1. Direct Workplace Impacts of the 2021 Heat Dome

Of the articles discussing the workplace impacts of the 2021 Heat Dome, the direct impacts (*n* = 311) affected 43 different occupations in five key ways. The most commonly cited impact was work cancellations or stoppages (*n* = 189) attributed to the severity of the heat. In particular, this impacted many construction crews, asphalt and concrete workers, and roofing teams. For example, one article interviewed a site supervisor of a roofing crew and reported that *“the temperatures at ground level may be uncomfortable but they’re downright dangerous if you’re working on a roof with no shade. The crew was pulled off its job installing cedar on a house. The guys are drinking two gallons of water a day but in this heat, they can only last 15 to 20 min before the heat gets to them”* [37].

Similarly, many articles discussed job cancellations for indoor workers, especially those who work in unairconditioned environments or near process-generated heat. For example, *“several businesses including restaurants in the [British Columbia] Valley closed temporarily to protect their staff from the extreme heat. Some food trucks recorded temperatures near 50 °C [122 °F] near their stoves”* [38]. For those unable to cancel jobs, work modifications (*n* = 120) were often reported in the media. Work modifications ranged from shortened workdays, shifts starting earlier in the day (i.e., before dawn), rotating crews more frequently, and arranging demanding work for lower heat periods, among other *“safety precautions to limit exposure during the high temperatures”* [39]. For example, one article reported that the heat was creating challenges for seasonal workers such as pavers and interviewed a Human Resources and Safety Manager who said, *“High temperatures have prompted the company to shorten workdays and start two hours earlier than the typical 7 a.m. to take advantage of lower temperatures in the morning. On Tuesday [June 29th, 2021], he said one 14-person crew laying hot asphalt was sent home shortly after 1 p.m. because of safety concerns and a lack of shade… It was becoming what we’ve considered hazardous”* [40].

Electrical utility workers were also commonly cited to be employing modified work strategies to combat the heat while trying to fix outages caused by infrastructure impacts related to the extreme heat (e.g., transformer burnouts from overheating) and the upsurge in air conditioning usage demand across the heat-affected region. For example, one article reported, *“BC Hydro appreciates that any outage can be concerning, but even more so in this extreme heat. Customers can be assured that crews are on standby and working as hard as they can to restore power quickly…However, the intense heat is adding to what is already an inherently dangerous job for crews. They must follow extra safety protocols due to the heat so in some cases power restoration is taking longer than normal”* [41]. Other less traditional occupations also experienced work modifications; for example, one professional soccer team moved their kickoff time after players, staff, and the medical team saw the forecast indicated 37 °C (99 °F) [42].

Another commonly mentioned direct workplace impact was heat-related strain (*n* = 102). In these articles, the strain caused by heat was often described by site representatives as making staff work *“under great personal discomfort”* [43] in what they considered *“hazardous”* [40] conditions, where crews were *“testing out cooling vests and following protocols like taking more frequent breaks from the sun and staying hydrated”* [40]. It was also noteworthy that one Union was quoted making a statement on worker heat protection: *“It has come to the Local’s attention that many of our members are working in temperature conditions which may be harmful to them. Specifically, we’ve heard that in several kitchens, our members are working in nearly 40 °C temperatures. This is unacceptable. We can’t accept it as a union and you do not have to accept it as a worker. You have the right to refuse”* [44]. Lastly, a few articles also provided warnings that due to the early timing of the extreme heat event in the summer season, *“physiological systems have yet to adapt to summer heat”,* and as a result, *“impacts to worker health is therefore expected to be considerable”* [45].

Although less commonly cited, a few articles also mentioned workers experiencing heat-related illnesses (or signs and symptoms indicative of a heat-related illness) (*n* = 3). For example, one article reported on a worker collapsing in the heat, *“a construction worker collapsed from the heat Monday, paramedics, firefighters, police and his fellow workers teamed up to get him down safely from the 21st floor, out of reach of an elevator”* [46]. Another article more broadly spoke of the physical toll on paramedics due to the additional donning of COVID-19 personal protective equipment, *“the paramedics said these shifts were… also physically exhausting—paramedics were wearing their uniforms as well as a plastic gown on top and other personal protective equipment while entering residences as hot as 45 Celsius to resuscitate people. They said they were soaked in sweat and some of their colleagues got sick from the heat”* [47].

Lastly, although no workers were reported in the media dataset to have died in Canada, a couple of articles did reference a workplace heat-related death (*n* = 2) that occurred in the Pacific-Northwest of the United States due to the same extreme heat event. For example, *“In the northwest United States, workers harvested blueberries in the middle of the night to beat the daytime heat; one farmworker died in an area where temperatures rose above 40 °C [104 °F]”* [48].

#### 3.2.2. Cascading (Indirect) Workplace Impacts of the 2021 Heat Dome

Numerous cascading (indirect) impacts (*n* = 492) were cited in the media concerning 45 occupations. Within these articles, workplaces were commonly impacted in four key ways. The most mentioned cascading workplace impact was an increased work demand (*n* = 428). The occupational group most implicated was healthcare providers (e.g., nurses, paramedics, dispatchers, home support workers, hospital staff, long-term care staff, and medical doctors). For example, many articles discussed *“increasing staff at hospital emergency centres to meet expected demand”* [49]. Another occupational group that experienced increased work demand was those in agriculture (e.g., farmers, fruit pickers). For example, one article reported that *“while city-dwellers struggled to survive the deadly heat, farmers across Western Canada have been desperately trying to save their cherries, wheat, and other crops. The high-stakes, thankless tasks—everything from midnight harvests to strictly rationing irrigation water”* [50].

Due to the low prevalence of air conditioning in British Columbia, air conditioner manufacturers, suppliers, and distributors were also reported to have experienced an uptick in demand. For example, one owner stated, *“The calls are coming in one after the next. We’re probably up double the volume from last year, especially with this upcoming week”* [51]. Although less dominant, other service industry workers at restaurants and retail establishments also experienced an increased work demand. For example, *“a manager said almost 500 customers were served [soft-serve ice cream] by mid-afternoon, and she expected to ‘get slammed’”* [52]. Additionally, due to other cascading weather events (e.g., wildfires), occupations involved in public safety (e.g., firefighters, park rangers) also experienced increased demand. For example, *“in an effort to reduce the fire risk to the park, which is extreme due to the current drought conditions and sustained heat events, park rangers will set up temporary overnight access control points”* [53].

In contrast, some occupations also experienced diminished work performance (*n* = 49). For example, one business owner discussed how the elevated overnight temperatures were impairing sleep quality and impacting his worker’s physical capacity during the day, *“It’s far too hot to have our staff in the fields or working over hot waffle irons…if staff aren’t sleeping, they’re going to be grinding through the day and not be functioning at a very high level*” [54]. Another cascading impact cited in the media related to the mental health implications (*n* = 48) of the extreme heat on workers was observed in reports of *“paramedics on stress leave and citizens asking—what went wrong?”* [55]. For example, one reporter spoke with four paramedics who worked during the heat wave and stated, *“all described a service overwhelmed with hundreds of high-priority calls. Oftentimes when they arrived at the scene, someone had already died. The paramedics said these shifts took an emotional toll and were also physically exhausting”* [47]. With the heat wave also burning crops across the region, many articles expressed concern *“about the mental health of producers”* [56]. Advocates were *“calling on the government to fund the creation of a 24/7 mental-health hotline for farmers”* [57] as *“the stress of this year is piled on top of the stress from the pandemic”* [56].

#### 3.2.3. Translational Workplace Impacts of the 2021 Heat Dome

A few translational impacts (*n* = 30) were also cited in the media to have affected seven occupations. The translational effects fell within four main themes. First, a few articles mentioned the loss of income (*n* = 21) concerning (or as a result of) the extreme heat event. For example, the heat significantly impacted the agricultural industry; one farmer reported that they *“were expecting to produce around 1000 pounds of currants at their hobby farm in B.C.’s Okanagan… But this summer, it got so scorching that…their raspberries melted to a hot, sticky goo, costing them thousands in lost revenue”* [58]. In some instances, these impacts were less explicit. For example, because of businesses closing, there is financial loss assumed, as illustrated by the following article excerpt: *“in the meantime, small towns are left to contend with how to navigate an uncertain future. Valemount’s only grocery store closed down unexpectedly because of the heat wave in late June”* [59]. Many of these articles also discussed financial impacts in the context of other coinciding crises. For example, *“the recovery time could be lengthy and costly… after B.C. cannabis farmers faced extreme heat and wildfires and as the industry recovers from COVID-19 shutdowns”* [60].

Similarly, some occupations were also reported to have experienced an increased financial burden (*n* = 5). For example, one canning business reported that *“price fluctuations due to weather are typical but nothing like the past year… we use fresh-pressed raspberry juice and that’s gone up 90%. That’s because most of our raspberries come from BC and obviously, they had the heat dome and that just killed the raspberry harvest”* [61]. Some of these translational impacts were also presented in the context of the ongoing COVID-19 pandemic; for example, it was reported that for cherry farmers *“the situation remains dire as the heat has compounded a pandemic-related labour shortage. That’s almost doubled the rate pickers can charge for their work compared to last year—but the price farmers receive for their cherries has barely budged”* [50].

In contrast, economic gain (*n* = 2) was identified for two occupations, air-conditioning installers and tanning salons, due to an uptick in business. For example, one article stated that *“heating, ventilation, and air conditioning installers are slowly re-cooping losses incurred as temperatures soared”* [51]. Lastly, job security (*n* = 2) was also an identified translational impact for agricultural labourers. For example, one article reported that *“peak blueberry and cherry season should be approaching, but some farmers are already pulling workers from the fields for the season”* [62]. Another article also cited that many pickers have been left out of work, with one farm owner saying, *“I’ve never left fruit on the tree…we’re just worrying about how everyone is going to survive. Every night, we almost cry. How are they going to feed their family? How are they going to pay their expenses? I wish we could do something”* [63].

## 4. Discussion

To help elucidate the spectrum of workers and workplaces impacted by extreme heat events in Canada, we conducted a systematized review and content analysis of thousands of media articles published on the 2021 Heat Dome. The findings identified 52 occupational groups that were reported in the media to have been impacted in some capacity by the extreme heat, including both traditionally heat-exposed outdoor workers and workers in unairconditioned indoor environments. Our analysis also highlighted that the extreme heat event impacted workers in different capacities, with some experiencing direct impacts, and others experiencing cascading and/or translational impacts, or a combination thereof. Thus, the findings broaden our understanding of the existing and growing threat that prolonged hot weather events have on workers’ health, safety, and productivity across an array of sectors.

### 4.1. Existing and Emerging At-Risk Sectors

Our media content analysis supports previous findings that show that many outdoor workers, such as asphalt and concrete workers [64], construction workers [2,65,66], electrical utility workers [67,68], miners [4,69,70,71], military [72], roofing contractors [73], and agricultural labourers (e.g., feed crop farmers, livestock farmers, fruit pickers) [2,74] experience impacts from working in extreme heat. These labour groups align with the traditional understanding of workers at risk of heat stress as they are often required to work in challenging ambient conditions, while wearing personal protective equipment, and/or while performing labour-intensive tasks [2,3,4,5].

The findings also extended our understanding of the range of outdoor occupations that can be impacted by extreme heat, including individuals like carnival workers and mail couriers, among others. Although these groups would also be commonly exposed to challenging outdoor ambient conditions, the extreme temperatures achieved during the heat event likely placed these workers at an even greater risk. For example, during the 2021 Heat Dome, over 1000 daily maximum temperature records were broken, including 103 all-time heat records and the highest recorded temperature in Canadian history (49.6 °C (121.28 °F) on 29 June 2021) [30]. Therefore, regardless of the intensity of work or uniform requirements, the heat posed a significant occupational and public health and safety challenge. Furthermore, during an extreme heat event, there is often little reprieve from the heat as nighttime lows remain elevated, as was seen with the 2021 Heat Dome [30], and thus workers receive reduced respite and recovery opportunities from the heat. As shown in the literature, consecutive workdays in the heat can lead to a progressive deterioration in an individual’s ability to dissipate heat [8], which has significant occupational health, safety, and productivity implications [1,9]. Therefore, all sectors that operate in the heat as a standard practice, regardless of work intensity or personal protective equipment, require modified management strategies specific to safe operation during extreme heat events.

Extending the emerging sectors further, the media articles also highlighted numerous indoor occupations impacted by the extreme heat, including healthcare staff, retail workers, museum staff, educators, and fitness instructors, among others. This is a noteworthy finding, as we identified primarily non-industrial indoor work environments. In contrast, the current literature on indoor occupational heat stress has focused mainly on workplaces with process-generated heat, such as foundries [75] or smelters [76,77]. In place of the risk posed by process-generated heat, indoor workers were described by the media to be impacted as a result of operating in unairconditioned environments. As the indoor climate is significantly influenced by the outdoor ambient temperature [78,79], this highlights the growing threat of extreme temperatures on indoor overheating, especially in unairconditioned environments [80]. This finding depicting the growing risk of indoor overheating was also corroborated by the recent release of data from British Columbia, which indicated that 35% of heat stress compensation claims in 2021 were for indoor workers (115 accepted heat stress claims; *n* = 74 outdoor workers, *n* = 41 indoor workers) [81]. Although not workplace-specific, the British Columbia Coroners Service also reported that high indoor temperatures were the primary cause of injury and death during the 2021 Heat Dome, with nearly 98% of deaths occurring indoors (*n* = 606), further emphasizing the threat of indoor overheating [32]. Thus, developing guidance to help protect individuals working in unairconditioned indoor environments during extreme heat events should be prioritized. For example, the current heat-health messaging statements released alongside heat warnings in Canada that are disseminated in the media could be revised from ‘people working outdoors’ [27] to include wording reflective of the risk posed to all workers exposed to indoor and outdoor heat. Furthermore, these findings support that although heat is not a *new* occupational hazard, it is a *new* exposure for some sectors and workers. In a recent article on climate change and occupational health and safety hazards, Schulte et al. [2] similarly reflected that “*the hazards will be known ones but in new situations*” (p. 193). Therefore, efforts should be focused on the prevention and control of the associated adverse effects for both the existing and emerging at-risk sectors, and the effective communication of these strategies via the media [82].

### 4.2. Impacts of the 2021 Heat Dome on Workers

Overall, most of the media content included in this analysis was related to the cascading (indirect) impacts of the extreme heat event, as opposed to the direct or translational impacts. This is interesting to note, as it enforces that the media-based narrative prioritized content such as increased work demand over information on the adaptive strategies employed to protect workers (e.g., implementing work-to-rest regimens, alternative task assignments) or the direct health impacts of the extreme heat. Notably, the recent release of the WorkSafeBC compensation claims data indicated that 115 claims for heat stress were accepted in 2021, which means a 180% increase over the previous three-year average (41%) [81]. Despite this significant increase, only three articles described stories of workers actually experiencing or displaying the signs and symptoms of heat-related illness, and only two articles described heat-related worker mortality based on a death in the United States related to extreme heat. This perhaps indicates a disconnect between the media outlets and the occupational health and safety system, which prevents or challenges the ability to report on workplace heat stress events promptly. Therefore, efforts should be made to ensure more timely communication of the threat posed by extreme heat to worker health occurs via the mass media.

### 4.3. Perspectives

The media is a vital information source for the public when preparing and responding to extreme heat events [25], and given the forecasted increase in the intensity and frequency of deadly heat in Canada because of climate change [11], communicating heat-health protective actions to the public is increasingly important [2,26]. However, based on our findings, only a few articles provided strategies for reducing the occupational health impacts of heat. For example, one article shared a series of stories from a cook, a road construction site flagger, and a power plant control mechanic on the heat in their workplaces. At the end of the article, a few lines were included which outlined the signs and symptoms of heat exhaustion and heat stroke, as well as the statement, “*risk can be reduced by limiting exposure to the sun wherever possible, drinking lots of water, wearing the right clothes, and taking rest breaks in cool, well-ventilated areas*” [29]. Including similar tailored statements among articles and news stories could be a simple yet effective strategy to disseminate heat-health protective actions. This avenue of education would be particularly valuable for employees within sectors or workplaces without comprehensive heat management plans (e.g., work-to-rest regimes, acclimatization programs, environmental monitoring, etc.), and thus would provide them another avenue for educating themselves on heat-health protection strategies applicable on and off the job site. Furthermore, in doing so, workers may become more knowledgeable on how to take action to protect their own health while also becoming more empowered to advocate for their rights within their workplace, such as the right to refuse unsafe work if the heat in the working environment becomes unmanageable.

Upon reviewing the media coverage, it also became apparent that we can leverage this information to gain a deeper understanding of areas where actions can be adjusted, improved, or revised to enhance the protection of worker health. For example, the flagger in the news article referenced above shared, “*It’s nearly impossible not to overheat, especially when working in full protective gear on sweltering highways with no proximity to shade…There’s no way you can have enough water to last you the day…The one thing that people never realize is our hard hats we have to wear—they are essentially a greenhouse on your head. You can take it off some days and your hair is just dripping, it’s wet with sweat. I throw ice cubes in my hard hat but they’re gone in like two minutes*” [29]. Company- and agency-based occupational health and safety managers can benefit from these types of stories, as they show the need to consider alternative strategies to employ during extreme heat events to adequately protect workers. Schulte et al. [2] recently shared a similar finding and discussed that in many cases, OHS programming for heat stress does not provide adequate protection for workers during extreme heat events because it often fails to account for the physiological limits of the body to adjust to rapid rises in temperature and its inability to sufficiently dissipate heat within hotter environments. Thus, the authors proposed that employers should implement heat alert plans to increase precautions taken to protect workers during extreme heat events [2]. For example, workplaces may consider employing more robust heat mitigation strategies or layering in additional protections such as wearable phase-change cooling materials that release/absorb sufficient energy at phase transitions to provide useful cooling [83], or strategizing water deliveries and shade provision when situationally appropriate. We propose to extend Schutle et al.’s [2] recommendation further, adding that the media can then also play a role in communicating this information to the public. As mentioned above, the role of the media in disseminating heat-health messaging is critical, especially for the emerging at-risk sectors that are not accustomed to climate-related hazards like extreme heat events [2].

### 4.4. Limitations and Future Work

In this study, we analyzed articles from the mass media; therefore, we were only able to explore the content as provided. Thus, we were not able to explore other factors that impact occupational heat stress, such as characteristics that influence how individual workers experience heat physiologically (e.g., age, sex, fitness) [84]. Furthermore, the articles did not present material related to other important aspects of OHS and heat management, such as the added vulnerability experienced by migrant workers or those performing precarious work (or work under non-standard employment). Therefore, future work should seek to explore other avenues and data sources that can provide insight into the impacts experienced by these subgroups of the workforce. It is also important to note that our analysis was limited to exploring Canada’s media-based communication landscape and does not reflect every North American region affected by the 2021 Heat Dome; the experiences in the United States could have been different. Future work may seek to explore how different OHS jurisdictions interact with the media during extreme heat events to help communicate heat-health protection efforts.

## 5. Conclusions

This study provides novel insights into the occupational health and safety impacts experienced during a historic extreme heat event in Canada. By conducting a systematized review and content analysis of media coverage about the 2021 Heat Dome, we were able to identify the occupations reported to have been impacted by the extreme heat event and detect the nature of these impacts. This unique approach to studying occupational health and safety allowed us to use stories of lived experience and testimonials of workers to shed light on critical challenges facing workers, while looking beyond the traditional claims data analysis method. In doing so, our findings illuminate the array of sectors at risk and a variety of different workplace impacts of heat. These findings demonstrate opportunities to improve occupational health communication related to extreme heat in the mass media—including encouraging media outlets to assist in increasing awareness of their entire readership, which includes workers, employers, decision-makers, and the public, about the significant risks and strategies that are needed to protect the health of all workers. With rising global temperatures and the increasing frequency and intensity of extreme heat events, climate change will have an increasingly detrimental effect on workplaces worldwide [1,2], and will directly threaten the health, safety, and well-being of billions of workers [85]. Therefore, occupational heat-health communication must be strengthened, as it is vital not only for worker education and empowerment, but also for drawing attention to the critical need of employers, workplaces, governments, and other stakeholders to dedicate the necessary resources to prioritize and protect worker health.

## Figures and Tables

**Table 1:** Occupations impacted by the 2021 Heat Dome in Canada, as identified in the media. The occupations are listed in order of frequency, where *n* = the number of articles referencing that occupation, followed by the number of references total within those articles. For example, if one article mentions Hotel Staff three times, it would be represented by (*n* = 1, 3).

Occupations Impacted by the 2021 Heat Dome in Canada as Identified in the Media		
Paramedics (*n* = 222, 444) Nurses (*n* = 92, 112) Educators (*n* = 89, 120) Firefighters (*n* = 89, 148) Farmers and Fruit Pickers (e.g., apples, berries, cannabis, cherries, Christmas trees, feed crops, grains, livestock, shellfish) (*n* = 86, 147) Police Officers (*n* = 79, 150) Dispatchers (*n* = 57, 89) Social Workers (*n* = 50, 66) Restaurant Staff, Servers, and Owners (*n* = 40, 77) Retail Staff (*n* = 34, 43) Bus Drivers (*n* = 31, 40) Hospital Staff (*n* = 31, 42) Hotel Staff (*n* = 26, 33) City Staff (*n* = 22, 37) Coroners (*n* = 21, 29) Air Conditioner Manufacturers, Suppliers, and Distributors (*n* = 20, 39) Electrical Utility Crews (*n* = 16, 33)	Mining and Metallurgical Workers (*n* = 13, 18) Museum and Heritage Site Staff (*n* = 13, 18) Farmers Market Vendors (*n* = 11, 15) Fitness Instructors (*n* = 11, 16) Curbside Collection Staff (*n* = 10, 13) Construction Workers (*n* = 9, 12) Asphalt and Concrete Workers (*n* = 7, 11) Medical Doctors (*n* = 7, 7) Professional Athletes (*n* = 7, 12) Insurance Analysts (*n* = 6, 10) Long-Term Care Staff (*n* = 6, 9) Nursery and Garden Centre Workers (*n* = 6, 11) Outreach Workers (*n* = 6, 6) Military Personnel (*n* = 5, 5) Taxi and Ride Sharing Drivers (*n* = 5, 6) Park Rangers (*n* = 4, 4) Railway Patrols (*n* = 4, 5)	Grocery Store Employees (*n* = 3, 3) Lawyers and Legal Service Providers (*n* = 3, 3) Telecommunication Crews (*n* = 3, 6) Carnival Workers (*n* = 2, 2) Home Support Workers (*n* = 2, 3) Landscapers (*n* = 2, 2) Mail Couriers (*n* = 2, 4) Researchers (*n* = 2, 2) Roofing Contractors (*n* = 2, 2) Bank Employees (*n* = 1, 1) Electricians (*n* = 1, 1) Emergency Support Services Volunteers (*n* = 1, 1) Forestry and Logging Workers (*n* = 1, 1) Outdoor Camp Counsellors (*n* = 1, 1) Outdoor Equipment Renters (*n* = 1, 1) Pharmacists (*n* = 1, 2) Psychologists (*n* = 1, 1) Tanning Salon Operators (*n* = 1, 2)

## Data Availability

Data are available from the corresponding author (Glen P. Kenny, gkenny@uottawa.ca, upon reasonable request and signed access agreement.

## Supplementary Materials

The following supporting information can be downloaded at: https://www.mdpi.com/article/10.3390/healthcare11172423/s1, Table S1. Direct, cascading, and translational workplace impacts of the 2021 Heat Dome as portrayed in the Canadian media.
