# Supplementary material for: Working under the 2021 Heat Dome: A Content Analysis of Occupational Impacts Mentioned in the Canadian Media
Source: Healthcare (Basel). 2023 Aug 30;11(17):2423. doi: 10.3390/healthcare11172423 (PMC10487058; doi:10.3390/healthcare11172423)
Supplement: Supplementary file 1 [file healthcare-11-02423-s001.zip › healthcare-2538931-supplementary.pdf]

healthcare

MDPI

Table S1. Direct, cascading, and translational workplace impacts of the 2021 Heat Dome as portrayed in the Canadian media.

Impact Classification	Type of Impact	Description of Impact	Occupations Impacte
Direct Impacts (n=311)	Work Cancellation or Stoppage (n=189)	Media content that describes the preventative cancellation of work to limit staff exposure or work stoppages that were led by either an employee, supervisor, union or safety agency as a direct result of the extreme heat.	Asphalt and Concrete Workers, Bank Employees, Bus Drivers, City Staff, Construction Workers, Coroners, Courthouse Staff, Curbside Collection Staff, Educators, Electrical Utility Crews, Farmers, Farmers Market Vendors, Fitness Instructors, Fruit Pickers, Grocery Store Employees, Landscapers, Mail Couriers, Miners, Museum Staff, Nursery and Garden Centre Workers, Nurses, Outdoor Camp Workers, Outdoor Equipment Renters, Restaurant Staff, Retail Staff, Roofing Contractors, Telecommunication Crews
	Work Modification (n=120)	Media content that describes workplaces modifying work procedures, including (but not limited to) altering work schedules to perform duties during cooler periods, rotation of staff, implementation of work-to-rest regimes, or alternative task assignments as a direct result of the extreme heat.	Asphalt and Concrete Workers, City Staff, Construction Workers, Curbside Collection Staff, Educators, Electrical Utility Crews, Emergency Support Services Volunteers, Farmers, Firefighters, Fitness Instructors, Fruit Pickers, Hospital Staff, Long-Term Care Staff, Mail Couriers, Nurses, Outreach Workers, Paramedics, Pipeline Crews, Police Officers, Railway Patrols, Restaurant Staff, Social Workers, Taxi/Uber Drivers, Telecommunication Crews
	Heat-Related Strain (n=102)	Media content that described the physical or psychological (mental) challenge faced by workers operating in the heat, including mentions of discomfort, hazardous operating conditions, and the demand on the thermoregulatory system as a direct result of the extreme heat.	Air Conditioner Manufacturers/Suppliers/Distributors, Asphalt and Concrete Workers, Bus Drivers, Carnival Workers, City Staff, Construction Workers, Curbside Collection Staff, Dispatchers, Electrical Utility Crews, Emergency Support Services Volunteers, Farmers, Farmers Market Vendors, Firefighters, Fruit Pickers, Grocery Store Employees, Long-Term Care Staff, Nurses, Outreach Workers, Paramedics, Pipeline Crews, Police Officers, Researchers, Restaurant Staff, Retail Staff, Roofing Contractors, Social Workers
	Heat-Related Illness (n=3)	Media content that described workers experiencing or displaying the signs or symptoms of a heat-related illness (e.g., heat cramps, heat rash, heat exhaustion, heat stroke) as a direct result of the extreme heat.	Construction Workers, Firefighters, Paramedics
	Heat-Related Death (n=2)	Media content that described worker mortality as a direct result of the extreme heat.	Fruit Pickers
Cascading (Indirect) Impacts (n=492)	Increased Work Demand (n=428)	Media content that described an increase in work demand, including (but not limited to) an above-average workload, increase in the number of jobs (e.g., being on call), increase in hours of work (e.g., overtime), changes to job requirements and scope (e.g., firefighters performing medical first response), or shift alterations (e.g., creation of additional shift rotations to meet client needs) as a secondary result of the extreme heat.	Air Conditioner Manufacturers/Suppliers/Distributors, Asphalt and Concrete Workers, Bus Drivers, City Staff, Construction Workers, Coroners, Curbside Collection Staff, Dispatchers, Educators, Electrical Utility Crews, Electricians, Emergency Support Services Volunteers, Farmers, Farmers Market Vendors, Fitness Instructors, Firefighters, Fruit Pickers, Grocery Store Employees, Health Service Navigators, Home Support Workers, Hospital Staff, Hotel Staff, Insurance Analysts, Law Enforcement Officers, Lawyers,

			Long-Term Care Staff, Medical Doctors, Military Personnel, Museum Staff, Natural Gas Technicians, Nursery and Garden Centre Workers, Nurses, Outreach Workers, Paramedics, Park Rangers, Pharmacist, Professional Athletes, Psychologists, Restaurant Staff, Retail Staff, Social Workers, Tanning Salon Owners, Telecommunication Crews
	Diminished Work Performance (n=49)	Media content that described diminished worker performance (e.g., slower work rates, lower productivity, increase in work errors) as a secondary result of the extreme heat.	Curbside Collection Staff, Farmers, Loggers, Restaurant Staff
	Mental Health (n=48)	Media content that described mental health impacts, including (but not limited to) stress leave requests, psychological trauma from witnessing significant death or increases in eco-anxiety and depression.	Coroners, Dispatchers, Farmers, Medical Doctors, Paramedics, Retail Staff
	Decreased Work Demand (n=1)	Media content that described workplaces that experienced lower work demand due to factors such as a diversion of services because of the extreme heat.	County Peace Officers
Translational Impacts (n=30)	Loss of Income (n=21)	Media content that described workplaces or workers that experienced income loss, such as workplaces that were forced to close or stop production or workplaces that experienced product damage/loss (e.g., crop loss) because of the extreme heat.	Farmers, Farmers Market Vendors, Restaurant Owners
	Increased Cost (n=5)	Media content that described workplaces that experienced added expenses because of responding to the extreme heat, such as industries that experienced labour shortages and increases in service rates or price fluctuations due to other industries' losses.	Farmers
	Economic Gain (n=2)	Media content that described economic gain due to an increase in service need and demand (e.g., increase in air conditioner service calls).	Air Conditioner Manufacturers/Suppliers/Distributors, Ice Cream Shop Owners, Tanning Salon Owners
	Job Security (n=2)	Media content that described employees that experienced job insecurity as a result of the cancellation or shortage of work available due to the extreme heat.	Fruit Pickers

(Note: As the articles were coded to multiple occupational impact categories where applicable, the indicated count values are greater than the total number of articles, n=705).
